# Novel RFID Multi-Label Network Location Measurement by Dual CCD and Non-Local Means–Harris Algorithm

**DOI:** 10.3390/s25020426

**Published:** 2025-01-13

**Authors:** Lin Li, Zhixin Jin, Junji Li, Zelin Zhang

**Affiliations:** 1 School of Electronic Information Engineering, Taiyuan University of Science and Technology, Taiyuan 030024, China; lilin001@tyust.edu.cn (L.L.); lijunji@tyust.edu.cn (J.L.); 2 College of Physical Education, Taiyuan University of Science and Technology, Taiyuan 030024, China; 3 Peking University Yangtze River Delta Institute of Optoelectronics, Nantong 100871, China; zhangzelin@ydioe.pku.edu.cn

**Keywords:** measurement, CCD, RFID, image processing, golden section, Harris corner detection

## Abstract

To improve the performance of Radio Frequency Identification (RFID) multi-label systems, the multi-label network structure needs to be quickly located and optimized. A multi-label location measurement method based on the NLM–Harris algorithm is proposed in this paper. Firstly, multi-label geometric distribution images are obtained through a label image acquisition system of a multi-label semi-physical simulation platform with two vertical Charge-Coupled Device (CCD) cameras, and Gaussian noise is added to the image to simulate thermoelectric interference. Then, a fast NLM algorithm that optimizes the kernel coefficient acquisition speed is used for image denoising. Finally, the Harris corner algorithm is used to obtain the corner points of the images. After screening the diagonal points, the pixel coordinates of the preset origin and the four corners of the labels are obtained. Furthermore, the actual coordinates of the labels are obtained according to the pixel relationship. The results show that the average absolute errors of x, y, and z coordinates are 0.773 mm, 0.782 mm, and 0.807 mm, respectively. In addition, the relative errors are 1.659%, 2.260%, and 0.258%, which shows the high location accuracy of the multi-label network. It is of great significance to measure and optimize the performance of multi-label systems.

## 1. Introduction

In the field of the Internet of Things (IoT), RFID, as a wireless technology for automatic identification, is an important bridge to connect things [1]. The RFID system is mainly composed of readers, RFID tags, and data processing systems. Its main features are lightweight and markable, which can identify targets and obtain corresponding data through radio frequency signals without human intervention [2,3]. RFID technology can be widely used in warehousing and logistics [4], intelligent transportation [5], and product retail [6,7,8]. However, in the actual complex application environment, RFID exposes problems such as short communication distance and reading ability, and still has many technical challenges in practical application.

RFID technology has undergone significant development in recent years, particularly in protocol design, antenna technology, and collision handling. ISO/IEC 18000-63 is a communication protocol for ultra-high-frequency (UHF) RFID tags, which has been extensively researched and optimized. In collision prevention mechanisms, many scholars have proposed time-domain collision methods, such as the ALOHA [9] and binary tree algorithms [10]. The ALOHA algorithm has advantages in some application scenarios due to its simplicity and low implementation cost, but is limited by low throughput and channel utilization. The binary tree algorithm is more efficient in data storage and retrieval, but may require more computing resources and complex implementation. In terms of efficiency optimization, Hu et al. proposed the TDMA method based on time slots, which enables multiple tags to respond safely at the same time and reduces the probability of conflicts [11]. The multiplexing technology of antennas has shown excellent performance in improving coverage and anti-interference capabilities. The multiplexing technology (MIMO) enhances the capacity and transmission efficiency of RFID systems through antenna channels. Khan et al. optimized signal reception through the spatial utilization of antennas, allowing multiple signal streams to be transmitted simultaneously in the same frequency band, thereby improving the throughput of RFID systems in high-density environments [12].

The development of RFID systems mainly focuses on improving anti-interference capabilities, collision prevention, and signal optimization. The ISO/IEC 18000-63 protocol has been widely used and has improved performance through various scheduling, encoding, and algorithm optimizations. Meanwhile, the multiplexing and MIMO technology of RWD antennas have shown great potential in increasing system capacity, improving transmission rates, and reducing interference. However, in practical application environments with complex electromagnetic wave interference, these methods cannot meet the real-time requirements and are no longer applicable. Yu et al. studied the performance optimization of multi-tag RFID and found that it is directly related to the geometric distribution of tags [13]. Therefore, by changing the geometric distribution of multiple labels, the reading performance of multiple labels can be optimized, and the anti-collision ability during the multi-label recognition process can be reduced.

In view of this, in order to obtain the optimal geometric distribution to achieve performance optimization of multi-label systems, a multi-label performance optimization system based on semi-physical simulation is proposed [13]. Zhang et al. proposed recognizing and matching label images from different camera perspectives [14]. The main process involves using two vertical cameras to collect and record images of the geometric distribution of multiple labels, denoising and feature location of the image, and obtaining the three-dimensional (3D) coordinates of labels in a multi-label system. Then, this set of coordinates would be stored in the dataset as a multi-label network. In addition, the multi-label performance of a multi-label network is described by the maximum read distance of the multi-label system, which is measured by the laser ranging sensor at the moment when the reader cannot receive the label information [15,16]. The above process is repeated to obtain lots of multi-label networks and their corresponding longest read distances. When the data collection is sufficient, the optimal multi-label geometric distribution is obtained through an optimization algorithm such as SVM.

From the above operation process of multi-label performance optimization systems based on semi-physical simulation, it can be found that obtaining 3D coordinates of labels through image processing is a very critical part of the optimization process which directly affects the accuracy of optimization. Therefore, this paper proposes a 3D network optimization method for RFID labels based on a fast NLM denoising algorithm with an improved kernel coefficient and the Harris corner detection algorithm. The method is divided into three parts: fast NLM denoising by the improved coefficient of the kernel function, detecting label grayscale coordinates using the Harris corner algorithm, and locating the 3D coordinates of labels based on the geometric optical similarity principle.

Among them, the denoising algorithm is essentially responsible for the pre-processing of the location algorithm. Its role is to remove the interference of thermoelectricity in the image so that the location algorithm can make the most of the useful information of the image to accurately locate the 3D coordinates of the RFID labels. The fast NLM denoising algorithm with an improved kernel function coefficient is an image-denoising algorithm formed after the integrating graph and modified kernel function were introduced to the original NLM algorithm [17,18]. Moreover, the optimal kernel function coefficient of RFID labels is obtained through the function graph. Under this algorithm, the PSNR of the operating graph is improved by at least 1.7561 dB and the average SSIM is increased by nearly 0.0141. This paper will use the golden section method instead of the function graph method, that is, the golden section point as the interval elimination test point, to further improve the NLM kernel function coefficient optimization time. This denoising algorithm will lay a solid foundation for the subsequent RFID labels’ three-dimensional coordinate location.

The Harris corner detection algorithm is an image detection algorithm that is widely used in various fields [19,20,21]. The huge difference between RFID labels and the surrounding environment makes the cornering algorithm able to accurately capture the coordinates of the calibration origin and the four corners of the label’s rectangle, to realize the location of the entire label in pixel space. The Harris corner detection algorithm can capture the coordinates of the calibration origin and the four angles of the rectangle labels. Compared with previous research, which needed to capture coordinates separately and use a template of labels to match other labels [22], our method is simplified in structure and reduces the complexity of the experimental process.

This paper has the following innovation points: First, the optimization time of the NLM kernel function coefficient is optimized by the golden section method to further improve the algorithm speed based on the fast NLM denoising algorithm of the improved kernel function coefficient. Second, a set of RFID multi-label 3D coordinate locations based on image processing are designed, which have considerable accuracy. In the future, we will provide conditions for finding the optimal distribution of the best performance of the multi-label network.

This paper is arranged as follows: Section 2 designs the algorithm process, and gives the basic principles of the fast NLM denoising algorithm, golden section, and Harris corner detection algorithm with an improved kernel function coefficient. Section 3 carries out specific experimental verification and in-depth analysis of the experimental results. Section 4 presents the conclusions of this study.

## 2. Algorithm Design and Basic Principle

### 2.1. Design of the Overall Algorithm

**Figure 1:**
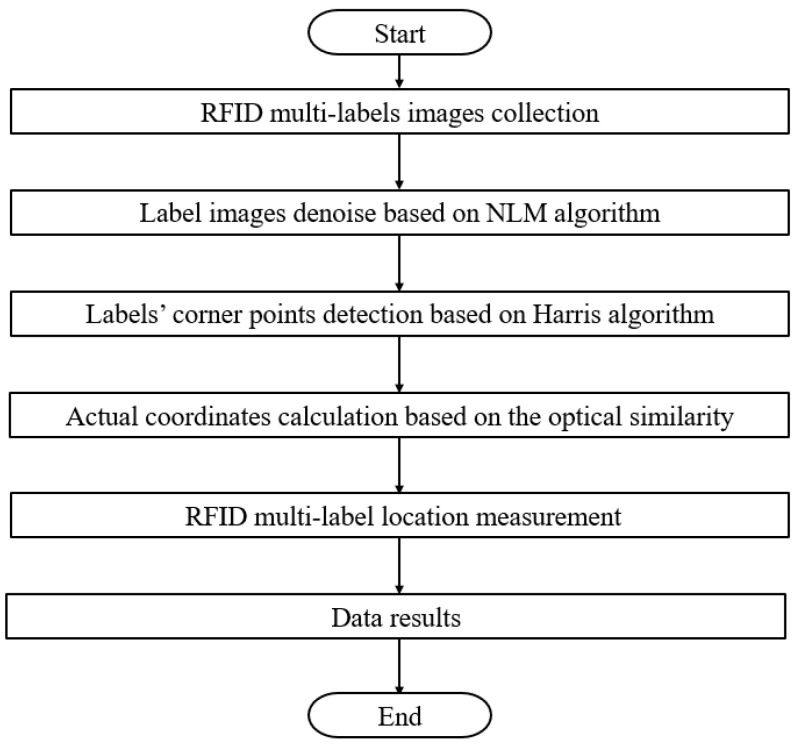
The specific flow chart.

The overall process of the RFID multi-label location measurement in this paper involves collecting RFID multi-label images by using the two vertical CCDs, and then using the improved NLM to denoise the images to improve the image quality. After that, Harris detection is used to locate the corners required by the RFID multi-label network. Finally, the actual coordinates of each RFID label are calculated according to the similarity principle of geometric optics to determine the location of the RFID multi-label network. The specific flow chart is shown in Figure 1. In the designed RFID hardware-in-the-loop simulation system, two CCD cameras are used to obtain RFID multi-label images, the multi-label images are denoised by the fast NLM denoising algorithm with improved kernel coefficients, and then the improved Harris corner detection method is used to detect the detection points of the tags. Finally, the actual coordinates of the RFID tag are calculated from the coordinates of the multi-tag corner detection by using the principle of geometric-optical similarity, and then the measurement of the three-dimensional geometric distribution of the multi-tag network is completed, and the three coordinate values of each tag are output.

### 2.2. The Fast NLM Denoising Algorithm with Improved Kernel Coefficient

**Figure 2:**
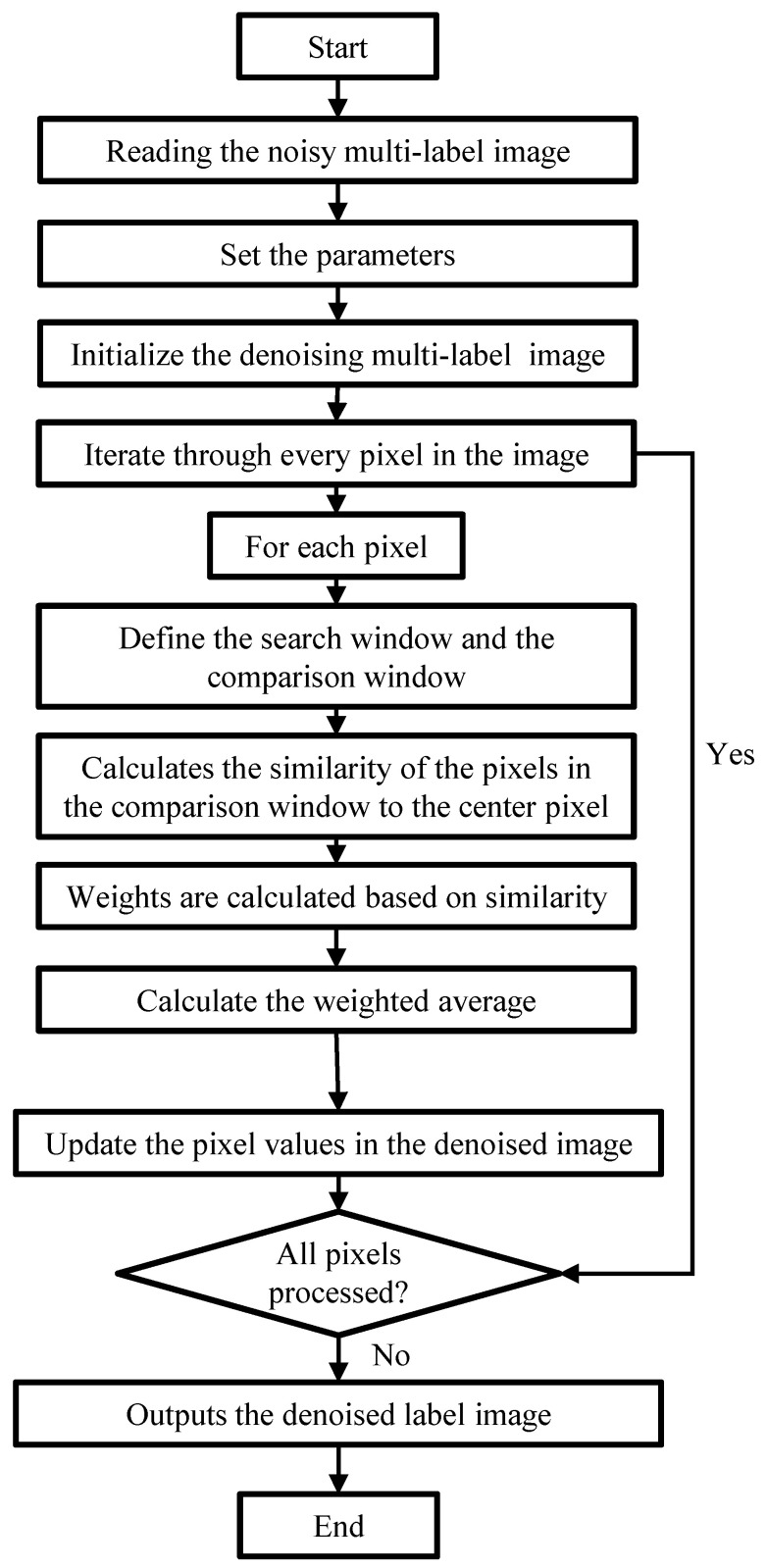
The NLM flow chart.

The principle of NLM is to determine the weight of the search window through the similarity between image pixels according to the kernel function of the whole image as a range, to achieve the purpose of image denoising. A detailed NLM flow chart is shown in Figure 2. To be specific, the fast NLM denoising algorithm with an improved kernel coefficient fuses the integral graph and the modified kernel function on the original NLM denoising algorithm and optimizes the denoising effect of the RFID label image by modifying the kernel function coefficient.

The kernel function of the fast NLM denoising algorithm with an improved kernel function coefficient is shown in Equation (1):

(1) $$\left\{\begin{array}{c} \omega \left(i,j\right)=\frac{1}{Z\left(i\right)}\exp\left(\frac{-{\left\|{Gv}_{x}-{Gv}_{y}\right\|}_{2}^{2}}{h^{2}}\right)\\ Z\left(i\right)=\sum_{j}\exp\left(\frac{-{\left\|v_{x}-v_{y}\right\|}_{2}^{2}}{h^{2}}\right) \end{array}\right.$$

where $\omega \left(i,j\right)$ is the similarity weights between pixels *i* and *j*, which determines pixel *j* to pixel *i*’s contribution. The greater the weight, the greater the influence of pixels *j* to *i*. *v_x_* and *v_y_* are a local window or feature area of an image that is calculated from the grayscale value of the image. $Gv_{x}$ and $Gv_{y}$ are the gradient of the image in different directions or the local features after filtering. $\|Gv_{x}-Gv_{y}{\|}_{2}$ is the Euclidean distance between $Gv_{x}$ and $Gv_{y}$ (L2 norm). The greater the distance, the greater the local regional difference. *h* is the smoothing parameter that controls the decay rate of the weights. A small *h*-value may cause the similarity to decay faster, and a large *h*-value may attenuate the similarity more slowly. *Z(i)* is the normalization constant, which is used to ensure that the sum of the weights *ω*(*i*,*j*) is 1 to avoid numerical instability. Grayscale values are converted into gradient information in the image, and this gradient information determines the similarity measure between different pixels.

The integral graph method is constructed as follows:

(2) $$\left\{\begin{array}{c} S_{t}\left(i,j\right)=\sum_{z\leq i,z\leq j}s_{t}\left(z\right)\\ s_{t}\left(z\right)={\left\|v\left(z\right)-v(z+t)\right\|}^{2} \end{array}\right.$$

where *V* (*z*) and *V* (*z + t*) represent two different fields. When calculating the distance between two neighborhoods, the integration graph is used to calculate the similarity without traversing all the pixels in the two neighborhoods to calculate the difference, which greatly improves the speed of completing the similarity calculation. The method of using the integration graph to calculate the similarity is shown as follows:

(3) $${\left\|v_{x}-v_{y}\right\|}_{2}^{2}=\frac{1}{d^{2}}S_{t}\left(i+d,j+d\right)+\frac{1}{d^{2}}S_{t}\left(i-d-1,j-d-1\right)\;\frac{1}{d^{2}}S_{t}\left(i+d,j-d-1\right)-\frac{1}{d^{2}}S_{t}\left(i-d-1,j+d\right)$$

where *d* is the edge length of the search window.

The optimal kernel function coefficient can be obtained by drawing the function relation diagram of the kernel function coefficient h with *SSIM* and *PSNR* [11], and the highest point of the function is the optimal kernel function coefficient. Specific algorithms of *SSIM* and *PSNR* are shown as follows:

(4) $$\left\{\begin{array}{l} MSE=\frac{1}{mn}\sum_{i=0}^{m-1}\sum_{j=0}^{n-1}{\left[I\left(i,j\right)-K\left(i,j\right)\right]}^{2}\\ PSNR=10\cdot \log_{10}\left(\frac{255^{2}}{MSE}\right)\\ SSIM=\frac{\left(2\mu_{x}\mu_{y}+c_{1}\right)\left(2\sigma_{xy}+c_{2}\right)}{\left(\mu_{x}^{2}+\mu_{y}^{2}+c_{1}\right)\left(\sigma_{x}^{2}+\sigma_{y}^{2}+c_{2}\right)} \end{array}\right.$$

where *MSE* is the mean square error, *µ_x_* and *µ_y_* as the means are used for the estimation of brightness, *σ_x_* and *σ_y_* as the standard deviation are used for the estimation of contrast, and *σ_xy_* as the covariance is used as the measure of structural similarity. *SSIM* ranges from 0 to 1. The larger the *SSIM*, the higher the image quality.

**Figure 3:**
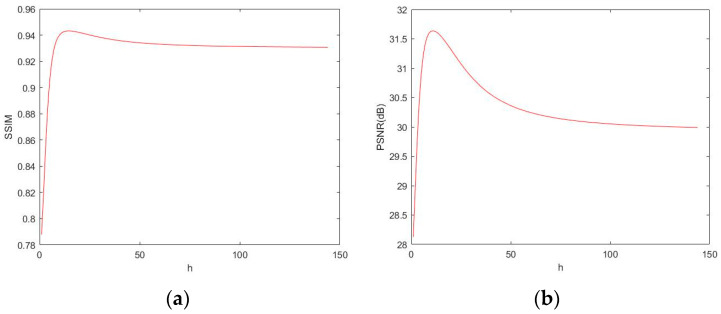
Function diagrams. (**a**) The function diagram between PSNR and h; (**b**) the function diagram between SSIM and h.

For the *RFID* label images, the functional relation diagram of the kernel function coefficient H with *SSIM* and *PSNR* is shown in Figure 3, and the optimal h obtained is 11.

### 2.3. Golden Section Method

**Figure 4:**
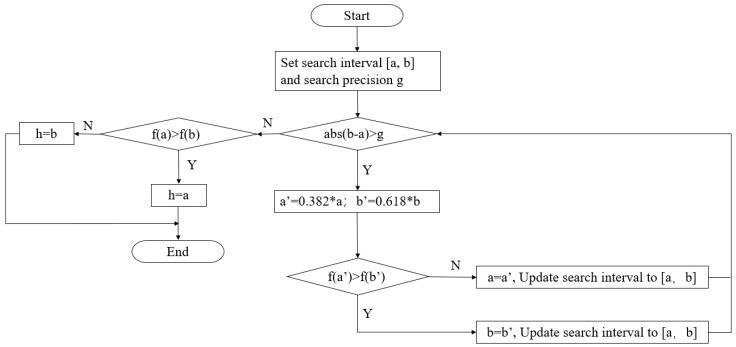
The flow chart of the golden section algorithm.

The golden section method is the development of the interval elimination method. By taking the golden section point as the trial point, it gradually reduces the search interval to obtain the extreme value of the single-peak function. As shown in Figure 3, the numerical relationship between h, *SSIM*, and *PSNR* is presented in the form of a single-peak function, which indicates that it is feasible to shorten the optimization time of h by using the golden section method. The algorithm flow of the golden section method for function optimization is shown in Figure 4.

In this study, a = 1, b = 140, and f is the SSIM or PSNR; the specific effect of the algorithm will be detailed in the next chapter.

### 2.4. Harris Corner Detection Algorithm

The basic principle of the Harris corner detection algorithm is to judge corner points, edges, and smooth areas in images by calculating the gray difference between two adjacent pixels. First, the matrix *H* is constructed:

(5) $$H=\sum_{x,y}w(x,y)\left[\begin{array}{cc} I_{x}^{2} & I_{x}I_{y}\\ I_{x}I_{y} & I_{y}^{2} \end{array}\right]$$

where *W(x*, *y)* is a two-dimensional Gaussian distribution, and *I_x_* and *I_y_* are the gray gradients of the image. There are two eigenvalues *λ*_1_ and *λ*_2_ in the matrix H, and let the corner criterion *R* be:

*R* = *λ*_1_*λ*_2_ − *k*(*λ*_1_ + *λ*_2_)^2^ (6)

where *k* = 0.04. If the maximum of *R* in the whole image is *R_M_*, it is stipulated that the corresponding *R* of a point is greater than the *R* of the eight neighboring points and 0.01 RM is the corner point.

## 3. Experiment and Analysis

**Figure 5:**
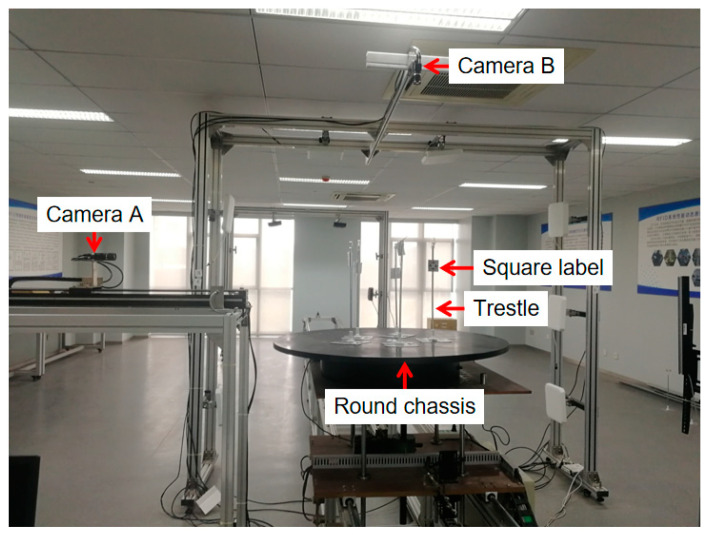
The physical configuration of the RFID semi-physical simulation system.

The research platform of this paper is the RFID semi-physical simulation system, and its physical configuration is shown in Figure 5. The system has two main functions: one is to obtain the furthest reading distance of labels by a laser rangefinder; the other is to obtain geometric distribution images of multiple labels by using two vertical cameras. The research in this paper only involves the image acquisition part, which is mainly composed of two vertical cameras A and B, a round chassis, RFID tags, and a trestle.

Two industrial CCD cameras are used in the mutually perpendicular multi-label imaging system to obtain image information. The light reflected by the measured label is refracted by the lens on the CCD sensor plane to generate an analog current signal. Then, it is converted into digital information through an A/D converter. Finally, it is transmitted to the computer through the communication interface of the industrial CCD cameras. When the camera is fixed, the blur of the image comes from the loss of multi-tag motion, light, and signal transmission. The relative motion between the moving label and the lens is an important factor affecting image quality.

**Figure 6:**
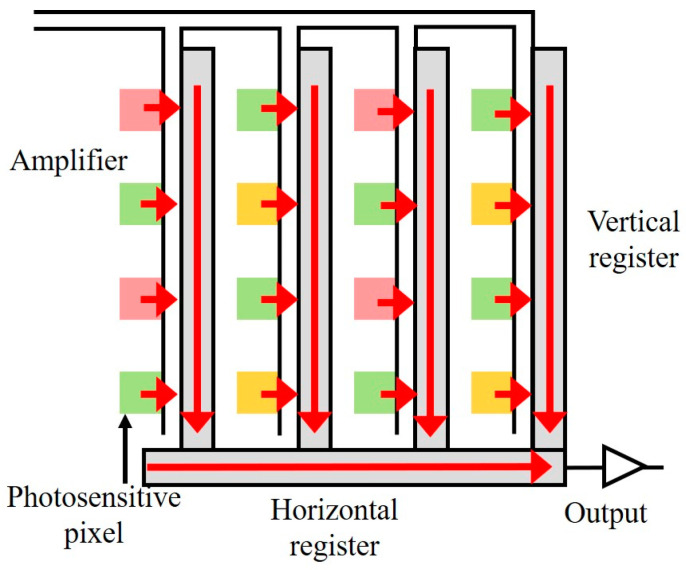
Operating principle of CCD chip.

A CCD is a semiconductor device, which determines the imaging quality of the camera. The more units, the higher the number of pixels, and the better the imaging quality. The CCD is the most commonly used image sensor in machine vision at present. It integrates photoelectric conversion, charge storage, charge transfer, and signal reading. It is a typical solid-state imaging device. The outstanding characteristic of the CCD is that it takes charge as a signal, unlike other devices, which take current or voltage as the signal. The working principle of CCD chips is shown in Figure 6. The red arrow indicates the direction of charge transfer. A CCD camera is composed of an optical lens, timing and synchronization signal generator, vertical driver, and A/D signal processing circuit. As a functional device, a CCD has the advantages of no burn, no lag, low-voltage operation, and low power consumption compared with vacuum tubes. In the CCD sensor, after receiving the light, the photosensitive image point is directly output to the vertical register and transferred to the horizontal register, and finally a unified output can be formed. Due to the weakness of the electrical signal generated by the photosensitive element and the voltage loss generated in the process, the analog-to-digital conversion cannot be completed. Therefore, the amplifier is required to amplify the electrical signal, and then it is processed through the A/D converter, and output to the DSP processing chip in the form of a decimal digital winter image matrix. Finally, the signal is compressed by the microprocessor and stored in a specific image file format. When the camera is fixed, the blur of the image comes from the loss of multi-tag motion, illumination, and signal transmission. The relative motion between the moving label and the lens is an important factor affecting the image quality.

In this paper, the first step is to carry out the RFID tag image-denoising process; the denoising algorithm used is the golden section method to accelerate the kernel coefficient optimization of the improved fast NLM algorithm. To verify the effectiveness of the golden section method, we compared its running time with the function diagram method and sequential interval elimination method. The results are shown in Table 1. The search intervals of the three methods are all [0, 140]. According to the sequential interval elimination method, the coarse search length is 10, and the fine search length is 1. The accuracy of the golden section method is set to 0.5. The final optimization results of the three methods are 11. It can be seen from Table 1 that the golden section method greatly improves the speed of optimizing kernel function coefficients.

The overall denoising effect of the proposed method is compared with that of the original NLM, fast NLM, NLM with improved kernel function, and NLM without kernel coefficient optimization. The specific method involves randomly extracting 200 different RFID label images from the image library of the RFID semi-physical simulation experiment system, adding Gaussian white noise to them to simulate thermoelectric interference (because the thermoelectric interference affects the image quality of the system more easily than other interference), and then using the original NLM, Fast NLM, NLM with improved kernel function, and NLM without kernel function coefficient optimization to denoise each RFID label image. Multi-label images contain noise interference encountered in the process of actual acquisition and transmission of images by the RFID hardware-in-the-loop simulation system. Therefore, it is feasible to use Gaussian white noise to model the actual acquired images. The effect of denoising is evaluated by calculating the SSIM and PSNR of the denoising image and the running time t of the denoising algorithm after obtaining the optimal kernel function. The final results are shown in Table 2. It can be seen from Table 2 that the denoising algorithm adopted in this paper is far superior to other NLM denoising algorithms both in quality and speed.

**Figure 7:**
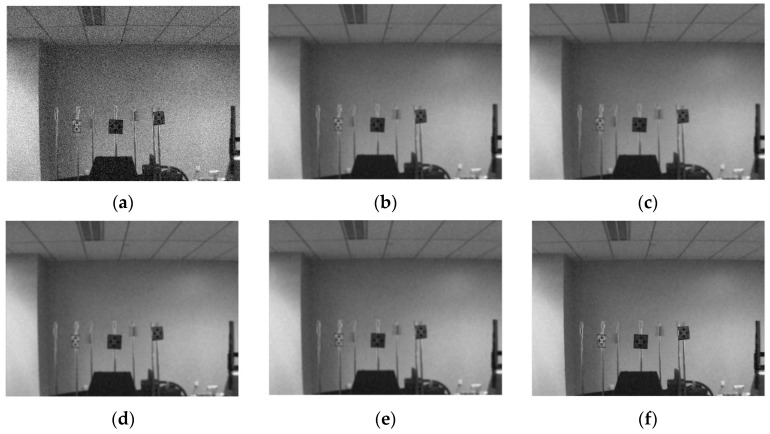
Denoising effect diagram. (**a**) Noisy image; (**b**) NLM; (**c**) FastNLM; (**d**) improved NLM; (**e**) improved FastNLM; (**f**) our algorithm.

To visually display the visual effect of the denoising algorithm in this paper, the denoising image of one of the label images processed by each denoising algorithm is presented in Figure 7.

**Figure 8:**
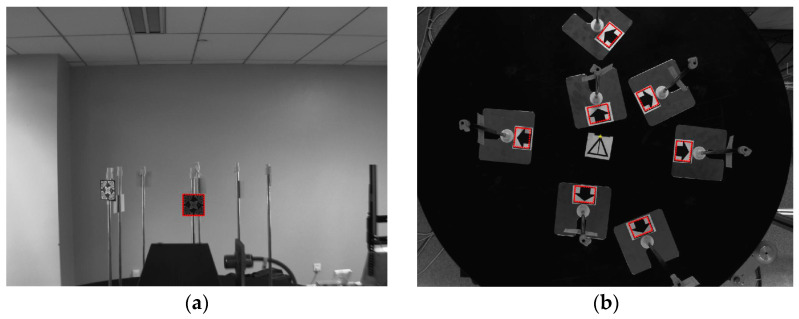
The images captured by the two cameras. (**a**) The image captured by camera A; (**b**) the image captured by camera B.

After denoising, this paper locates the pixel coordinates of two images containing the same RFID multi-label geometric distribution information collected by two cameras via the Harris corner detection algorithm. For the image collected by camera A, this paper needs to capture the whole label. For the images collected by camera B, what this paper needs to capture are the marker points. Figure 8 is the effect diagram after the Harris corner detection algorithm is run and irrelevant points are screened out. To observe the capture effect intuitively, some points are connected with dotted lines in this paper. The red box in (a) and (b) represents the capture result of label distribution, and the yellow point in (b) represents the capture result of the origin of calibrated coordinates. It can be found that the algorithm captures the labels and marker points well.

The following is the location of multi-label network coordinates. This algorithm realizes the transformation from pixel coordinates to spatial coordinates, which requires the 3D coordinates of one label and the z coordinates of another label as reference values. The pixel coordinates collected by a label camera B are denoted as (*a*, *b*), the pixel y coordinates collected by camera B are denoted as c, the actual 3D coordinates are denoted as (*a*′, *b*′, *c*′), the *z* coordinate of another label pixel is denoted as *d*, and the actual *z* coordinate is denoted as *d*′. The transformation formula of pixel coordinates to spatial coordinates is shown in Equation (7):

(7) $$\left\{\begin{array}{c} x=\frac{a\prime }{a}(x_{0}-n)\\ y=-\frac{b\prime }{b}\left(y_{0}-m\right)\\ z=d\prime +\frac{c\prime -d\prime }{c-d}(z_{0}-d) \end{array}\right.$$

where (*x_0_*, *y_0_*, *z_0_*) is the 3D pixel coordinates of the label to be located, and (*x*, *y*, *z*) are the actual 3D coordinates of the label after location. (*n*, *m*) is the pixel coordinate of the origin, and the specific value is (0.440, 0.628). (*a*, *b*) is (0.495, 0.925), and *c* is 0.751; the coordinates of (*a*′, *b*′, *c*′) are (0.204, 0.038, 0.220). All values are in m.

Through the above calculation process, coordinates of 110 groups of multi-label distribution are located. The coordinate results of the located label networks are shown in Table 3. For the complete 3D coordinate data, see https://download.csdn.net/download/u010493489/90236740, accessed on 26 November 2024.

To evaluate the location effect of the proposed method, the mean absolute error r and mean relative error r’ are used to describe the location effect of this experiment. Its definition is as follows:

(8) $$\left\{\begin{array}{c} r=\frac{\sum_{i=1}^{n}\left|h-h_{0}\right|}{n}\\ r^{\prime }=\frac{\sum_{i=1}^{n}\frac{\left|h-h_{0}\right|}{h_{0}}}{n} \end{array}\right.$$

The error analysis of the location results of the RFID label networks is shown in Table 4. The average absolute error of *x* coordinates is 0.773 mm and the average relative error is 1.659%. The mean absolute error of *y* coordinates is 0.782 mm and the mean relative error is 2.260%. The mean absolute error of *z* coordinates is 0.807 mm and the mean relative error is 0.258%. The average absolute error is less than 1 mm, and the average relative error is less than 3%. It is proved from the data that the error of this method is small, which meets the requirement of labels’ 3D coordinate location in the RFID semi-physical simulation experiment.

## 4. Conclusions

In order to quickly locate and optimize the multi-label network structure in complex environments, this paper proposes the RFID multi-label location measurement method based on the NLM–Harris algorithm. The method is composed of an image-denoising algorithm based on a fast NLM algorithm with an improved kernel function coefficient and an RFID multi-label coordinate location algorithm based on the Harris corner detection algorithm. At the same time, the coefficient optimization part of our NLM algorithm is accelerated by the golden section method. Finally, by locating 110 groups of RFID multi-label networks with different distributions, the average absolute error and average relative error of coordinates in the RFID multi-label networks are calculated. In the laboratory environment, the average absolute error and average relative error obtained are 1 mm and 3% at most. The data volume of RFID multi-label network coordinates ensures the stability and reliability of the calculated absolute error and relative error, so we can prove that the method has high location accuracy in the multi-label network. In the future, we will continue to conduct further experiments and introduce deep learning for deblurring analysis of multi-label images. Furthermore, modeling and analyzing RFID systems in complex environments, and using visual intelligence to measure the position of multi-label, can further improve the reading performance of multi-label. In addition, for the dynamic control of reading/writing devices, we will consider how to adjust the device’s working mode according to different environmental channel conditions (such as interference and attenuation) and the number of tags, optimize read/write efficiency, and especially determine how to avoid collisions and improve recognition accuracy in high-density environments.

## Figures and Tables

**Table 1:** Iteration times and optimization times of the three kernel function optimization methods.

Algorithm	Number of Iterations	*t/*s
Function diagram method	140	668.0836
Sequential interval elimination method	57	170.9005
Golden section method	12	115.2156

**Table 2:** The SSIM, PSNR, and t of different denoising algorithms.

Algorithm	SSIM	PSNR (dB)	*t/*s
NLM [8]	0.9259	29.8026	194.7913
FastNLM [12]	0.9256	29.7731	3.3360
Improved NLM [13]	0.9274	29.8349	193.9935
Improved FastNLM	0.9272	29.8101	3.2632
Our Fast NLM	0.9415	31.5910	3.4289

**Table 3:** The coordinate location results of label networks.

Number	*x*_1_/m	*y*_1_/m	*z*_1_/m	*x*_2_/m	*y*_2_/m	*z*_2_/m	· · ·	*x*_7_/m	*y*_7_/m	*z*_7_/m
1	0.207	0.017	0.426	0.154	−0.193	0.311	· · ·	0.091	0.119	0.290
2	0.204	−0.038	0.292	0.098	−0.227	0.426	· · ·	0.120	0.096	0.290
3	−0.070	0.254	0.281	−0.035	−0.084	0.351	· · ·	−0.035	−0.083	0.352
· . .	· . .	· . .	· . .	· . .	· . .	· . .	· · ·	· . .	· . .	· . .
109	0.069	0.289	0.325	−0.117	0.330	0.378	· · ·	−0.115	0.328	0.378
110	−0.162	0.069	0.346	0.061	0.172	0.269	· · ·	0.062	0.171	0.269

**Table 4:** Error analysis of RFID label networks’ location results.

Number of Labels		1	2	3	4	5	6	7	Average
*x*	*r*(mm)	0.766	0.759	0.831	0.812	0.769	0.730	0.746	0.773
	*r*′(%)	2.037	1.066	2.023	2.340	1.302	1.812	1.033	1.659
*y*	*r*(mm)	0.831	0.710	0.720	0.766	0.740	0.828	0.878	0.782
	*r*′(%)	1.494	3.027	1.616	1.998	2.988	2.000	2.694	2.260
*z*	*r*(mm)	0.821	0.818	0.769	0.871	0.802	0.746	0.818	0.807
	*r*′(%)	0.264	0.267	0.240	0.277	0.258	0.233	0.266	0.258

## Data Availability

Data are contained within the article.
